# Teachers as Gatekeepers in Adolescent Suicide Prevention: The Role of Suicide-Related Knowledge, Empathy, and Collaborative Self-Efficacy

**DOI:** 10.3390/bs16030409

**Published:** 2026-03-11

**Authors:** Federica Graziano, Chiara Davico, Irene Giordano, Elena Lonardelli, Daniele Marcotulli, Emanuela Calandri

**Affiliations:** 1 Department of Psychology, University of Turin, 10124 Torino, Italy; irene.giordano@unito.it (I.G.); emanuela.calandri@unito.it (E.C.); 2 Section of Child and Adolescent Neuropsychiatry, Department of Public Health and Pediatric Sciences, University of Turin, 10124 Torino, Italy; chiara.davico@unito.it (C.D.); elena.lonardelli@unito.it (E.L.); daniele.marcotulli@unito.it (D.M.)

**Keywords:** adolescence, suicide, teachers, secondary school, gatekeepers, suicide-related knowledge, empathy, self-efficacy, prevention

## Abstract

Teachers play a key role as gatekeepers in adolescent suicide prevention, and knowledge about suicidality is a well-established predictor of teachers’ gatekeeper self-efficacy. However, little attention has been paid to other potential predictors, particularly teachers’ empathy and self-efficacy in collaborating with colleagues to support adolescents experiencing mental distress. This cross-sectional study examined the associations between suicide-related knowledge, teacher empathy (perspective taking, empathic concern, and personal distress), collaborative self-efficacy, and gatekeeper self-efficacy. A convenience sample of 455 Italian secondary school teachers (84% female; mean age = 46.7 years, SD = 10.5) completed an anonymous self-report questionnaire. Data was analyzed using descriptive statistics and multiple regression analyses. Overall, teachers demonstrated adequate knowledge of adolescent suicidality. However, several myths persisted, including the belief that openly talking about suicide may increase risk. Higher gatekeeper self-efficacy was associated with greater knowledge, higher levels of perspective taking and empathic concern, lower levels of empathic distress, greater collaborative self-efficacy, and prior exposure to adolescent suicidality. These findings underscore the joint contribution of personal and relational factors to teachers’ gatekeeper self-efficacy and offer important implications for the development of teacher-focused gatekeeper training programs.

## 1. Introduction

Suicide is one of the leading causes of death worldwide; in 2021, more than 700.000 people took their own lives (WHO, 2025; Davis Weaver et al., 2025). Globally, suicide is the third leading cause of death in the 15–29 age group and the fourth among adolescents aged 15–19 years (WHO, 2025). In Europe, suicide is the second leading cause of death among young people aged 15–29 years, following unintentional injuries (EUROSTAT, 2025). In 2023, in Italy, there were 376 deaths by suicide in the 15–29 age group, accounting for 10% of the total number of suicides recorded in the general population (*n* = 3821) (ISTAT, 2025). In recent years, following the COVID-19 crisis, suicide rates have increased (Pathirathna et al., 2022; Marco et al., 2025); in Italy, for example, the female population aged 10–24 years was the most affected, with a notable increase especially in the adolescent age group between 15 and 19 years (Grande et al., 2025). Suicide is a complex phenomenon arising from the interdependence of biological, psychological, sociocultural, and environmental factors (WHO, 2014); none of these factors alone can provide a complete causal explanation. Given this multifactorial nature, the WHO (2021) emphasizes the importance of a multisectoral, innovative, and comprehensive prevention plan aimed at both reducing the global suicide mortality rate and improving the quality of life and biopsychosocial well-being of each individual. Suicide prevention in adolescence must therefore take into account the various risk and protective factors, as well as the contexts, most relevant to young people (Carballo et al., 2020).

In this context, schools are increasingly recognized as key settings for youth suicide prevention, given that young people spend most of their time at school (Ayer & Colpe, 2023; Laurenzi et al., 2023). “Gatekeepers” are individuals who have greater opportunities to identify situations of vulnerability and potential suicide risk at an early stage due to their professional or social position. Gatekeepers, including first responders, pediatricians, and school staff, can play a significant role in suicide prevention. In particular, teachers play a central role as gatekeepers in adolescent suicide prevention due to their frequent interactions with young people (Torok et al., 2019; Stickl Haugen et al., 2023). The literature has extensively documented the effectiveness of training programs for gatekeepers in suicide prevention. Such interventions promote the acquisition of essential operational skills, including recognizing risk signals, assessing critical situations, and referring individuals to professional help (Plöderl et al., 2023; Liu et al., 2025). To this end, training programs aim to raise awareness of suicide risk and knowledge of the phenomenon, as well as enhance gatekeepers’ self-efficacy and their relational and socio-emotional skills (Torok et al., 2019; Stickl Haugen et al., 2023).

Self-efficacy refers to an individual’s belief in their ability to select and execute behaviors necessary to achieve specific outcomes. This belief includes confidence in one’s ability to regulate motivation, behavior, and social environments (Bandura, 1997). In the context of suicide prevention, gatekeepers’ self-efficacy refers to the perception of being able to observe risky behaviors, communicate effectively with people in difficulty, and refer them to professional help services. Gatekeepers’ self-efficacy is an important predictor of whether a gatekeeper will actually intervene to help at-risk individuals (Kuhlman et al., 2017). Several studies have demonstrated that greater gatekeeper self-efficacy correlates with a better ability to translate perceptions into concrete actions, such as implementing effective supportive behavior and adopting appropriate, positive attitudes towards prevention (Mo et al., 2018; Kingi-Uluave et al., 2025).

Given the importance of gatekeeper self-efficacy, the literature has examined its predictors, focusing primarily on variables such as knowledge level, personal motivations, and attitudes toward suicide and its prevention. Possessing adequate knowledge, such as basic information about suicide and its risk factors, helps reduce the impact of myths and misconceptions about suicide. This knowledge can influence attitudes towards the phenomenon and prevent both the failure to recognize warning signs and the lack of adoption of effective coping behaviors (Schurtz et al., 2010; Arendt et al., 2018; Nicholas et al., 2020; Ayala Romera et al., 2024). Myths are common misconceptions about suicide based on false information (Moskos et al., 2004; WHO, 2014). Some of the most widespread myths include the belief that talking about suicide with a person at risk can encourage or incite suicidal behavior, the idea that suicide occurs without warning signs, and the belief that those who express suicidal thoughts do not really intend to take their own lives but are simply seeking attention. Such beliefs, which tend to minimize or underestimate the threat of suicide, may compromise accurate risk assessment and lead to the neglect of relevant signs. Individuals at risk express their intentions through multiple channels, most notably nonverbal communication and specific behaviors, such as giving away valuables or tidying their personal belongings (Ayala Romera et al., 2024). Recognizing the negative influence of these myths and misconceptions is key to tackling the stigma and critical issues surrounding mental health emergencies (Ayala Romera et al., 2024). Such beliefs fuel stigma, which in turn can discourage help-seeking, and promote social isolation, thereby increasing the risk of suicide (Wyllie et al., 2025). When faced with suicidal behavior, teachers may experience feelings of helplessness and guilt (Soja Santos et al., 2022), which can lead to uncertainty about their ability to intervene effectively.

Attitudes towards suicide prevention and behavioral intention to intervene are two additional factors that can influence gatekeepers’ responses in risky situations (Kingi-Uluave et al., 2025). A positive attitude and intention to help can facilitate the involvement of students in difficulty, whereas a negative attitude towards suicide prevention can increase reluctance to intervene (Ko, 2025). These factors can, in turn, be influenced by other elements, including stigma and cultural taboos surrounding suicide, as well as a low perceived competence in managing suicide risk situations (Borah et al., 2025). Therefore, increasing knowledge, promoting a positive approach to suicide prevention, and deconstructing myths that may hinder the willingness or ability to intervene, represent fundamental steps for teachers (Schurtz et al., 2010; Borah et al., 2025).

While knowledge, attitudes, and behavioral intentions are the factors most frequently investigated in the literature, other dimensions, such as empathy and self-efficacy in collaborating with colleagues, also have significant potential to enhance the ability to detect signs of suicidality among students, but remain relatively unexplored by researchers.

Empathy, defined as the ability to understand and share the emotional experiences of others, is a multidimensional construct comprising several closely interrelated dimensions. According to the Davis model (Davis, 1980, 1983), relevant dimensions of empathy include the following: empathic concern, the capacity to feel affective reactions of concern, sympathy, and compassion for people experiencing negative events; perspective taking, the ability to understand and adopt another person’s viewpoint; and personal distress, the perception of being overwhelmed by another person’s negative emotions (such as pain or sadness) to the extent that one absorbs the emotional experience and focuses on one’s own resulting discomfort. While perspective taking refers to the cognitive dimension of empathy, both empathic concern and personal distress refer to the affective dimension (Gilet et al., 2013; Toffol et al., 2025).

Empathy is a fundamental skill for teachers, as it enables them to understand their students’ emotional experiences and needs. This ability promotes the development of better educational relationships based on trust, support, and mutual understanding (Hen, 2020; Sun et al., 2023). Furthermore, an empathetic relational context promotes students’ resilience and supports their psychological well-being, creating a safer and more engaging learning environment (Aldrup et al., 2024; Ampofo et al., 2025).

Understanding and sharing students’ emotions can help identify signs of significant psychological distress and enable sensitive responses to their needs (Ampofo et al., 2025). Consistent with this perspective, the study by Ampofo et al. (2025) highlights that recognizing and responding appropriately to students’ emotional needs is associated with positive mental health outcomes, such as reduced levels of stress, anxiety, and depression. However, empathy is not always “positive”: being empathetic can lead to personal costs, such as suffering, emotional distress, and even burnout, especially when one is overwhelmed by the negative emotions of others (i.e., empathic distress) (Calandri et al., 2021). In these situations, the person may focus more on alleviating their own discomfort than on actually helping those who are suffering. High levels of affective empathy can therefore compromise teachers’ well-being and reduce their effectiveness in supporting students. Burnout, which has been extensively studied among teachers (Wink et al., 2021; Brandão et al., 2025), is also a significant risk factor for those who serve as gatekeepers. Dimensions of burnout (emotional exhaustion, depersonalization, and reduced personal accomplishment) can hinder the ability to recognize students’ needs or warning signs and to manage situations of distress effectively. In particular, burnout has been associated with reduced perspective-taking, increased focus on one’s own emotional exhaustion, and the use of defensive coping strategies to protect oneself from emotional overload, such as avoidance or emotional distancing (Brandão et al., 2025).

For this reason, it is necessary to consider the various dimensions of empathy—cognitive and affective—in their interdependence, especially when dealing with extremely complex issues such as adolescent suicide. However, the ability of gatekeepers to act effectively does not depend solely on the individual. As mentioned above, suicide is a complex phenomenon and therefore cannot be effectively addressed by a single professional. In this study, we considered collaborative self-efficacy as teachers’ perceived ability to mobilize collegial resources when addressing students’ mental health needs rather than managing such situations individually. Specifically, collaborative self-efficacy refers to teachers’ beliefs in their capability to engage effectively with colleagues to identify, discuss, and jointly address students’ mental health problems, including signs of psychological distress and suicidality. Collaborative self-efficacy can be considered a specific aspect of teacher self-efficacy, namely their confidence in managing instructional and classroom-related demands (Zee & Koomen, 2016; Lazarides & Warner, 2020). To our knowledge, in the gatekeeper literature, the teacher’s capability to collaborate effectively with colleagues has not been considered a distinct domain, although peer collaboration and support are recognized by teachers as resources for acting as gatekeepers (Nadeem et al., 2011). We therefore focused on this construct as a key factor to reinforce teachers’ ability to recognize and respond to risk, to activate a functional support network for the adolescent, and to contrast feelings of isolation when dealing with such serious issues (Davico et al., 2024b).

Developing a deeper understanding of the role played by these dimensions—knowledge, empathy, and collaborative self-efficacy—has significant practical implications for preventing suicide among adolescents, as it can help identify which teachers’ skills should be enhanced through training programs. Strengthening these skills can, in turn, improve the ability to recognize and manage risk situations in students.

In light of the literature examined, this study had two main aims:

1. To assess suicide-related knowledge and the endorsement of myths about suicidality in a convenience sample of secondary school teachers.
2. To investigate the role of teachers’ suicide-related knowledge, components of empathy (i.e., perspective taking, empathic concern, and personal distress), and collaborative self-efficacy on gatekeepers’ self-efficacy. We expected that higher gatekeeper self-efficacy would be associated with greater suicide-related knowledge, perspective taking, empathic concern, collaborative self-efficacy, and lower personal distress.

## 2. Materials and Methods

### 2.1. Participants and Procedure

The participants were selected from a convenience sample of 466 teachers consecutively recruited in a large study evaluating the feasibility of a theater-based gatekeeper training for the prevention of adolescent suicide (for a detailed description of the study, see Davico et al., 2024b). Teachers were recruited on a voluntary basis from secondary schools in the northwest of Italy. After providing informed written consent, participants completed an anonymous self-report questionnaire administered online via the Limesurvey platform. The study was reviewed and approved by the Bioethics Committee of the University of Turin (Italy) (Protocol number 0132972, 20 February 2024). The participants received no compensation for taking part in the study. Of this convenience sample of 466 teachers, 455 responses were considered valid for statistical analysis.

Most participants were female (82.6%, *n* = 376; 16.3% male, *n* = 74; missing 1.1%, *n* = 5), a trend that reflects the current sex prevalence of teachers in Italian schools (OECD, 2023). They were aged between 21 and 65 (*M_age_* = 46.7, *SD* = 10.5); 167 (36.7%) were working in middle schools (students aged between 11 and 13) and 280 (61.5%) in high schools (students aged between 14 and 19). The average years of teaching experience was 15.7 years (*SD* = 11.8, range 1–44 years). Most participants (95%) stated that they had never received specific training on suicide. With regard to exposure to suicide, participants reported significant direct or indirect experiences related to students and/or people belonging to their social network (family members, friends, or acquaintances) (Table 1).

### 2.2. Measures

#### 2.2.1. Gatekeeper Self-Efficacy

Gatekeeper self-efficacy is a 7-item scale used to evaluate teachers’ perceived ability to recognize warning signs of suicide, communicate with a student with suicidal thoughts, give help, and indicate appropriate health services. The questions were adapted from previous studies (Albright et al., 2016); questions regarding the perceived ability to detect signs of suicide, to communicate with students, and to provide help were not modified, whereas questions referring to specific mental health services offered in the United States context were reworded to refer to the Italian context. The scale showed good reliability in our previous study (Davico et al., 2024b). Each item was scored on a 5-point Likert scale (from 1 = Completely unable to 5 = Completely able; range 7–35) (e.g., “How capable do you feel to communicate appropriately with a student who shows signs of strong psychological distress?”). Reliability in our study was satisfactory (Cronbach’s alpha = 0.85) and exploratory factor analysis (EFA) suggested a one-factor solution explaining 52.5% of variance.

#### 2.2.2. Suicide-Related Knowledge

Suicide-related knowledge was evaluated using 9 true/false questions investigating knowledge of suicide rates among adolescents, main epidemiological data on adolescent suicide, myths and misconceptions about suicide (e.g., “asking whether the person has thought about suicide increases the likelihood of suicide”), risk and protective factors (e.g., “self-harm is a risk factor for suicide”), and main warning signs of suicide (e.g., “social withdrawal can be a warning sign for suicide”) (Davico et al., 2024b). The knowledge score was the sum of the correct responses (1 = correct/0 = incorrect), with higher scores indicating more knowledge (range 0–14).

#### 2.2.3. Empathy

Empathy was evaluated through a brief form of the Interpersonal Reactivity Index (Ingoglia et al., 2016). For the present study, three subdimensions were considered, namely Perspective Taking (PT) (e.g., “Before criticizing somebody, I try to imagine how I would feel if I were in their place”), Empathic Concern (EC) (e.g., “I often have tender, concerned feelings for people less fortunate than me”), and Personal Distress (PD) (e.g., “In emergency situations, I feel apprehensive and ill-at-ease”). Each subscale is composed of 4 items on a 5-point Likert scale ranging from 1 (it does not describe me at all) to 5 (it describes me very well). Reliability was satisfactory (PT Cronbach’s alpha = 0.82; EC Cronbach’s alpha = 0.81; PD Cronbach’s alpha = 0.78).

#### 2.2.4. Teacher Collaborative Self-Efficacy

A 4-item scale was purposefully developed to investigate teachers’ perceived ability in collaborating with colleagues to help a student manifesting signs of mental distress (Davico et al., 2024b). Each item was scored on a 5-point Likert scale (from 1 = Completely unable to 5 = Completely able; range 4–20) (e.g., “How capable do you feel to seek solutions together with colleagues when a student shows signs of psychological distress?”). Reliability in our study was satisfactory (Cronbach’s alpha = 0.90), and exploratory factor analysis (EFA) suggested a one-factor solution explaining 76.2% of variance.

### 2.3. Data Analysis

All statistical analyses were performed with IBM SPSS Statistics (version 29). After removing 11 records for which the percentage of missing data for the study variables exceeded 10%, the MCAR (Missing Completely at Random) test (Little, 1988) on the sample of 455 participants was not statistically significant, indicating that the missing data were completely at random. Therefore, imputation was performed using the EM (Expectation–Maximization) procedure. Descriptive analyses included frequencies, means, and Pearson’s bivariate correlations between the study variables. A hierarchical regression analysis was conducted to identify variables significantly associated with gatekeeper self-efficacy. Prior training and exposure to student suicidality were entered as control variables in the first step. Exposure to suicidality was calculated as the sum of exposure to suicidal ideation (yes = 1; no = 0), suicide attempt (yes = 1; no = 0), and suicidal act (yes = 1; no = 0), with scores ranging from 0 to 3. Correct suicide-related knowledge was entered in the second step, while empathy dimensions and collaborative self-efficacy were entered in the third step.

## 3. Results

### 3.1. Descriptives

The percentages of correct responses to suicide-related knowledge items showed that teachers generally had a good level of accurate knowledge, although some misconceptions persisted (Table 2).

Most teachers (81%) were aware of epidemiological data showing that suicidality is the second leading cause of death among young people aged 15–25. Almost all participants agreed that not only health professionals can talk about suicide (82.1%) and recognized the importance of sharing concerns about a student’s suicidal thoughts with colleagues (96.9%).

Although 62.6% of teachers correctly answered that the statement “If you want to end your life, you don’t seek help” was false, recognizing that a suicidal person might ask for help in non-verbal ways too, about a third of them (37.4%) still believed that someone who is serious about ending their life will not seek help. This might lead them to ignore or underestimate the significance of some warning signs. As for knowledge about risk factors for suicidality, while almost all participants (95.4%) recognized isolation as a risk factor, around 60% identified academic difficulties, mood swings, substance abuse, and self-harm. Notably, only one third recognized talking about suicide as a risk factor, and fewer than 15% indicated giving away one’s valuable possessions as a warning sign of suicidality.

### 3.2. Overview of Correlations Among Study Variables

Table 3 shows the correlation among the variables of this study. Results showed that prior suicide-related training was positively associated with gatekeeper self-efficacy (r = 0.102, *p* < 0.05) and exposure to student suicidality (r = 0.148, *p* < 0.01). The last variable was positively correlated with gatekeeper self-efficacy (r = 0.164, *p* < 0.01) as well as with age (r = 0.111, *p* < 0.01) and years of professional experience (r = 0.101, *p* < 0.05). Age and years of professional experience were negatively associated with suicide-related knowledge (r = −0.134, *p* < 0.01; r = −0.170, *p* < 0.01); age was also negatively correlated to collaborative self-efficacy (r = −0.093, *p* < 0.05). Suicide-related knowledge was negatively associated with personal distress (r = −0.129, *p* < 0.01) and positively correlated with collaborative self-efficacy (r = 0.111, *p* < 0.01). The latter was also positively associated with perspective taking (r = 0.228, *p* < 0.01) and empathic concern (r = 0.119, *p* < 0.01), but was negatively correlated with personal distress (r = −0.147, *p* < 0.01)

Finally, gatekeeper self-efficacy was positively correlated to prior training, suicide-related knowledge, exposure to student suicidality, perspective taking, empathic concern and collaborative self-efficacy (this being the strongest correlation observed with r = 0.476 (*p* = 0.01); it was negatively correlated to personal distress.

### 3.3. Results of Regression Analysis

In the first step, prior training and exposure to student suicidality were entered as control variables. Exposure to student suicide had a positive impact on gatekeeper self-efficacy (*β* = 0.156, *p* = 0.001), while prior training was unrelated to gatekeeper self-efficacy. In the second step, correct suicide-related knowledge resulted as a variable significantly associated with gatekeeper self-efficacy (*β* = 0.157, *p* = 0.001). The addition of empathy dimensions and collaborative self-efficacy in the third step produced an increase in explained variance (22,8%), bringing the final model to explain 27% of the variance in gatekeeper self-efficacy. In this model, collaborative self-efficacy was the variable most strongly associated with gatekeeper self-efficacy (*β* = 0.404, *p* = 0.001). Furthermore, prior exposure to student suicidality (*β* = 0.116, *p* = 0.01), better knowledge (*β* = 0.087, *p* = 0.05), and greater empathic concern (*β* = 0.117, *p* = 0.05) showed positive and significant effects, while personal distress showed a significant negative effect (*β* = −0.102; *p* = 0.05). Prior training instead was not significant (Table 4).

## 4. Discussion

The first aim of this study was to assess suicide-related knowledge and the endorsement of myths about suicidality in a convenience sample of Italian secondary school teachers. The second aim was to explore the role of teachers’ suicide-related knowledge, empathy, and collaborative self-efficacy on teachers’ perceived ability to detect warning signs of suicidality among adolescent students.

Regarding the first aim, our results generally showed correct and accurate suicide-related knowledge among study participants, although the endorsement of myths about suicidality still remained widespread.

Our findings reflect the increasing awareness of adolescent suicidality among teachers. Social isolation was widely recognized as a risk factor for suicidality, perhaps because it is an evident behavior clearly related to serious psychological problems. Nonetheless, knowledge about other risk factors was still lacking, especially when behaviors were less visible (e.g., self-cutting) or might be related to diverse causes (e.g., academic difficulties, mood swings, or substance abuse). The lack of knowledge about self-cutting as a risk factor might be partly due to the fact that adolescence is considered an evolutive period characterized by big emotional “storms”; self-harming behaviors could be thus perceived as an act that the adolescents performs simply to attract attention, rather than a warning signal to be taken seriously and addressed (Adhilah & Setiawati, 2025).

The endorsement of myths about suicidality remained widespread among our study participants. About one third of participants believed that “talking about suicide encourages suicidal behavior” while only half recognized that “talking about suicide can reduce the risk of suicidal behavior”. This result highlights the persistence of one of the most widespread suicide myths: many people believe they may trigger suicide plans by openly discussing suicidal thoughts with individuals at risk. On the contrary, such conversations are actually an important way to contribute to treatment and prevention (Arendt et al., 2018; Nicholas et al., 2020).

Another widespread myth is that “if someone wants to end their life, they do not seek help,” which was endorsed by about one third of teachers in our study. This reflects the mistaken belief that suicide occurs without warning signs. On the contrary, the literature highlights that most people who die by suicide have given some clue or warning (Schurtz et al., 2010; Arendt et al., 2018).

The fact that only one third of participants identified talking about suicide as a risk factor also reflects the myth that “individuals who talk about suicide will not actually do it.” In reality, overt communication of suicidal thoughts to others should always be taken seriously as a warning sign of suicidality.

As repeatedly pointed out, these myths are related to distorted knowledge that contributes to the stigmatization of suicidal individuals and hinders both help-seeking behaviors and intervention by community members and families (Niederkrotenthaler et al., 2010). By contrast, debunking suicide myths is associated with increased suicide-related knowledge, which in turn can promote behavioral intentions to provide help to suicidal individuals (Arendt et al., 2018; Borah et al., 2025).

Regarding the second aim of the study, some interesting results emerged from correlations.

Firstly, some considerations should be made regarding teachers’ exposure to adolescent suicidality. In our study, teachers who have been in contact with adolescents at risk of suicide or who have experienced suicide have had more training on suicide risk and possess greater self-efficacy as gatekeepers. This result is consistent with previous studies, which highlight that teachers exposed to student suicidality are more likely to increase their awareness of suicide risk, choose to attend prevention training, and develop greater competence in detecting warning signs of suicidality and supporting students (Kõlves et al., 2017; Stickl Haugen et al., 2023).

Nonetheless, the effect of exposure to student suicidality on teachers’ gatekeeper self-efficacy remains controversial; the traumatic experience of suicidal behavior may lead to decreased self-confidence and reluctance to intervene due to feelings of guilt and helplessness (Soja Santos et al., 2022; Stickl Haugen et al., 2023). The results of our study are consistent with research highlighting the positive relationship between prior exposure to student suicidality and higher gatekeeper self-efficacy. However, the impact of student suicidality (including suicide, suicide attempts, and suicidal thoughts) on various aspects of gatekeeper self-efficacy—such as recognizing warning signs, providing support, and referring to healthcare professionals—requires further investigation. Specifically, it would be interesting to examine whether there are different relationships between gatekeeper self-efficacy and exposure to suicide, on the one hand, and gatekeeper self-efficacy and exposure to suicidal thoughts and attempts, on the other.

Secondly, some considerations arise regarding the role of teachers’ years of experience, which were negatively correlated with correct knowledge and unrelated to gatekeeper self-efficacy. The negative association between years of teaching experience and correct knowledge about adolescent suicidality may seem counterintuitive; however, it can be understood in terms of generational and cohort-related differences in teacher training. Teachers with fewer years of experience are more likely to have completed pre-service education during a period when mental health promotion and suicide prevention received greater institutional and public attention (Anderson et al., 2019). In contrast, more experienced teachers may have been trained at a time when suicide was less openly discussed in educational contexts. This suggests gaps in teacher pre-service curricula and highlights the importance of continuous professional development. Although this speculation requires further research, our findings highlight the need for ongoing, up-to-date training in suicide prevention at all career stages.

The absence of relationships between teacher experience and gatekeeper efficacy is consistent with other studies in which years of teaching were not correlated with teachers’ comfort and confidence in identifying and supporting suicidal youth (Hatton et al., 2017). This result suggests that the general knowledge of more experienced teachers does not necessarily translate into gatekeeper self-efficacy, which requires specific training.

Results of the regression indicated that, after controlling for previous exposure to suicide and prior training, variables significantly associated with gatekeeper self-efficacy were accurate suicide-related knowledge, greater empathic concern, lower personal distress, and higher collaborative self-efficacy.

Regarding control variables, only previous exposure was related to gatekeeper self-efficacy, consistent with the literature (Stickl Haugen et al., 2023), while prior training was unrelated to gatekeeper efficacy, probably because only a minority of participants had received specific training.

Suicide-related knowledge is confirmed to be associated with higher gatekeeper efficacy, highlighting the central role of accurate information as a first step in feeling competent to recognize warning signs of suicidality and to deal with potentially suicidal individuals (Nicholas et al., 2020).

Beyond correct knowledge, both empathy and collaborative self-efficacy were related to higher gatekeeper self-efficacy. Regarding empathy, our findings highlight the specific role of the affective dimensions, though in opposite directions: higher gatekeeper efficacy was associated with greater empathic concern and lower personal distress.

On the one hand, teachers’ ability to understand and share students’ emotions can promote the recognition of psychological difficulties and suicidal signs, and in turn foster the adoption of helping behavior. On the other hand, teachers’ personal distress related to the emotional burden from contact with students’ negative emotions and psychological difficulties might hinder their ability to detect signs of deep suffering and to implement useful actions.

These findings are consistent with studies highlighting the complex and multifaceted role that empathy plays in student–teacher relationships (Hen, 2020; Sun et al., 2023; Aldrup et al., 2024; Ampofo et al., 2025).

Finally, the perceived ability to collaborate with colleagues in supporting students with serious psychological difficulties emerged in our study as the variable most strongly associated with gatekeeper self-efficacy. The perception of being able to collaborate with colleagues reduces the risk of teacher isolation and feelings of loneliness, the possibility of relying on peers, and the opportunity to activate a support network to address the serious issue of adolescent suicidality. In particular, the role of social support from colleagues has been highlighted in other studies on the effectiveness of gatekeeper training (Nadeem et al., 2011). Specifically, support received from coworkers has been shown to facilitate the application of acquired knowledge and skills and to promote gatekeeper behavior (Moore et al., 2011).

### 4.1. Implications

The study has relevant theoretical and practical implications. To the best of our knowledge, this is the first study to consider the role of diverse variables (i.e., not only teachers’ knowledge about suicidality but also empathy dimensions and perceived ability to collaborate with colleagues) on gatekeeper self-efficacy. The study highlights the relevance of both personal and relational factors for gatekeeper self-efficacy and emphasizes the complexity of the relationships among the variables considered.

The results of our study can usefully inform gatekeeper interventions targeting teachers. Promoting accurate suicide-related knowledge is the first step to increasing gatekeeper self-efficacy. Specifically, training should focus on increasing teachers’ awareness of risk factors for adolescent suicidality and their ability to detect signals that are often not clear or overt. Our findings showed that only 58% of participants recognized self-harm as a risk factor for suicide. This result indicates the need to raise awareness of Non-Suicidal Self-Injury (NSSI) as a major suicide risk. This is particularly relevant given that self-cutting has increased among today’s adolescents (Lim et al., 2019).

Moreover, it is imperative to debunk suicide myths, as recommended in international guidelines on how to talk about suicide and report news (WHO, 2023) and in previous studies (Davico et al., 2024a). Educating not only teachers but the entire community about suicide and providing accurate facts and knowledge can effectively contribute to reducing suicide myths. Increased knowledge, in turn, has been shown to have a positive effect on behavioral intentions to provide adequate help to suicidal individuals (Arendt et al., 2018).

Additionally, the results of our study highlighted the importance of addressing teachers’ emotional and relational competencies as core topics in gatekeeper training. Experiential training that promotes competencies such as empathy and self-efficacy for collaboration with colleagues in addressing adolescent suicidality is recommended. Therefore, psychoeducational training to increase accurate knowledge should be accompanied by experiential sessions, such as those based on role-playing and active learning. In this regard, the use of performing arts, such as theater, is recommended, as indicated in the literature and previous studies (Davico et al., 2022).

The proposed programs must focus on collaboration between teachers, as the social network among adults makes individual teachers more effective in preventing the risk of suicide and intervening when necessary.

### 4.2. Limitations

The study has some limitations. First, the sample consisted of individuals who might have had greater sensitivity, interest, or awareness of the topic. Therefore, the use of a convenience sample prevents generalization of the results. Although a large sample of secondary school teachers was involved, it was not representative of the population under study. Participation was voluntary and may have been biased by the topic of the study, as teachers more interested in suicidality or with prior exposure to suicidality were more likely to participate, as pointed out in similar studies (Hatton et al., 2017; Kõlves et al., 2017; Stickl Haugen et al., 2023). Further studies on larger and more representative samples are needed to reduce possible response bias. Second, the study participants were recruited from a large urban area in Northwest Italy, and further studies should include teachers from rural areas and different educational paths (e.g., lyceum or technical/vocational training school) to achieve greater representativeness. Third, the cross-sectional design of the study does not allow for conclusions about causal relationships between the variables considered. Bidirectional relationships between variables are likely, and only further longitudinal studies would allow for investigation of causal relationships. Fourth, our study focused only on gatekeeper self-efficacy and did not consider actual gatekeeping behaviors. Further research should therefore examine the role of teachers’ knowledge, empathy, and ability to collaborate with colleagues in the implementation of gatekeeper behavior. Previous research indicates that gatekeeper training does not always affect actual behavior (Torok et al., 2019; Robinson-Link et al., 2020). In light of our findings, it would be interesting to examine whether collaborative self-efficacy among teachers can promote not only gatekeepers’ self-efficacy but also their intention and actual behavior.

## 5. Conclusions

Adolescent suicidality is a significant public health concern, and an increasing number of studies have examined the importance of gatekeeper training involving teachers for the prevention of such tragic events. While accurate knowledge remains the first step in prevention, attention should also be given to fostering teachers’ emotional and relational competencies. For this reason, gatekeeper training should provide teachers with appropriate educational and psychological tools to address the growing psychological difficulties faced by adolescents. Given the central role teachers play in their daily interactions with adolescent students, these issues should be adequately considered not only when implementing specific gatekeeper training but also in the broader context of teacher pre-service and in-service education.

## Figures and Tables

**Table 1:** Characteristics of participants (N = 455).

	Participants
Biological sex, N (%)	
Female	376 (82.6)
Male	74 (16.3)
Missing	5 (1.1)
Age, *M* (*SD*)	46.7 (10.5)
School type, N (%)	
Middle school	167 (36.7)
High school	280 (61.5)
Missing	8 (1.8)
Years of professional experience, *M* (*SD*)	15.7 (11.8)
Exposure to student suicidality, N (%)	
Suicidal ideation	110 (24.3)
Suicide attempt	115 (25.4)
Suicide	35 (7.8)
Exposure to family members, friends or acquaintances suicidality, N (%)	
Suicidal ideation	148 (32.7)
Suicide attempt	153 (33.8)
Suicide	147 (32.5)
Prior suicide prevention training, N (%)	
Yes	23 (5)
No	428 (95)

**Table 2:** Percentages of correct responses to suicide-related knowledge items.

Item	Percentage of Correct Responses
1. Suicide is the second leading cause of death among 15–25 age group in Italy	81%
2. Asking someone if they have thought about or are thinking about suicide may cause the person to think about suicide	77.7%
3. If you want to end your life, you don’t seek help	62.6%
4. It is not possible to prevent suicide because it is an unpredictable event that occurs without warning signs or precursors.	96.9%
5. Only professionals can ask people if they have thought about or are thinking about suicide	82.1%
6. Asking someone if they have thought about or are thinking about suicide can reduce the risk of suicide	53.6%
7. People who self-harm are at greater risk of suicide	58.3%
8. If you are concerned that a student may be having suicidal thoughts, it is best to maintain confidentially and not discuss it with colleagues	96.9%
9. Which of the following are warning signs that someone is thinking about suicide? (select all options that you think are correct) (all correct)	
Sudden and repeated absences from school or decline in performance	57.4%
Sudden mood swings	64.2%
Substance abuse	56.3%
Giving away valuable possessions	14.1%
Isolation	95.4%
Talking about suicide	32.7%

**Table 3:** Descriptive statistics and correlations between study variables.

	Variables	*M* (*SD*)	1	2	3	4	5	6	7	8	9	10
1	Prior training	—	-									
2	Exposure to student suicidality	—	0.148 **	-								
3	Age	46.7 (10.5)	0.072	0.111 **	-							
4	Years of professional experience	15.7 (11.8)	0.083 *	0.101 *	0.783 **	-						
5	Gatekeeper self-efficacy	21.25 (3.56)	0.102 *	0.164 **	0.063	0.010	-					
6	Suicide related knowledge	9.29 (1.86)	0.034	0.079 *	−0.134 **	−0.170 **	0.154 **	-				
7	EMP: Empathic Concern	15.07 (3.27)	−0.016	0.024	−0.015	−0.035	0.171 **	0.048	-			
8	EMP: Personal Distress	8.57 (3.02)	−0.036	−0.088 *	−0.074	−0.033	−0.137 **	−0.129 **	0.304 **	-		
9	EMP: Perspective taking	13.57 (3.09)	0.044	−0.008	−0.071	−0.082 *	0.232 **	0.065	0.435 **	0.106 *	-	
10	Collaborative self-efficacy	14.08 (2.61)	0.025	0.071	−0.093 *	−0.053	0.476 **	0.111 **	0.119 **	−0.147 **	0.228 **	-

Note. EMP = Empathy. ** Correlation is significant at the 0.01 level (2-tailed). * Correlation is significant at the 0.05 level (2-tailed).

**Table 4:** Hierarchical regression analysis predicting gatekeeper self-efficacy.

	Gatekeeper Self-Efficacy					
	*B*	*SE B*	Confidence Intervals (95%)		*β*	*Adj R*^2^ (∆*R*^2^)
			Lower	Upper		
Step 1						0.028
Exposure to student suicidality	0.691	0.210	0.278	1.103	0.156 ***	
Prior training	1.125	0.774	−0.397	2.647	0.069	
Step 2						0.050 (0.024)
Exposure to student suicidality	0.639	0.208	0.230	1.048	0.144 **	
Prior training	1.071	0.766	−0.433	2.576	0.066	
Suicide relate-knowledge	0.298	0.088	0.124	0.472	0.157 ***	
Step 3						0.274 (0.228)
Exposure to student suicidality	0.516	0.183	0.156	0.875	0.116 **	
Prior training	0.876	0.671	−0.442	2.194	0.054	
Suicide-related knowledge	0.166	0.078	0.012	0.320	0.087 *	
EMP: Perspective taking	0.102	0.053	−0.002	0.206	0.088	
EMP: Empathic concern	0.127	0.051	0.026	0.227	0.117 *	
EMP: Personal distress	−0.119	0.051	−0.221	−0.018	−0.102 *	
Collaborative self-efficacy	0.548	0.058	0.434	0.662	0.404 ***	

Note. EMP = Empathy; * *p* < 0.05, ** *p* < 0.01, *** *p* < 0.001. First step F (2, 440) = 7.330, *p* < 0.001; second step F (3, 439) = 8.792, *p* < 0.001; third step F (7, 435) = 24.790, *p* < 0.001.

## Data Availability

Data that supports the findings of this study are available from the corresponding author upon reasonable request.
